# On the Causes of Irritability and Excitability

**Published:** 1803-07-01

**Authors:** M. De La Melhere


### *On the* Causes *of* Irritability *and* Excitability; *by* M. De La Melhere. *Communicated by our Correspondent at Paris.*

In the present state of human acquirements, this question is one of the most interesting in Physiology, but it is one of extremely difficult solution; with a view to lessen these difficulties, the author offers some reflections. The errors which we refute as well as those we commit, he observes, lead often to the truth. He begins by laying down certain facts. Irritable parts, he observes, taken out of the body, preserve for a considerable time their motion. The heart of a frog or of a turtle, will beat for an hour after separation from the animal; but even when these motions cease, they may be renewed for some instants by various means. 1st. By heat, the effect of which in reproducing the motions in the parts of animals, induced Goodwin to conclude that caloric was the principle of excitability. 2d. By light, which has the same effect as caloric; it is well known that animals kept long in the dark lose their energy and excitability. 3d. By oxygen, as well as every body which contains it. Humboldt has shewn that the heart of a frog, which had discontinued to beat, was again excited to action by plunging it in superoxygenated muriatic acid. If it be afterwards dipped in a sulphuret of potash its motions cease. Girtanner even pretends that oxygen is the principle of irritability. 4th. By Galvanism. 5th. By electricity. In order to discover, says the author, the causes of these phenomena, let us enquire into the nature of the substances which can act on the animal fibre; such as, 1. Heated bodies and caustics. 2. Moisture and dryness. 3. Galvanism and electricity. 4. The particular structure of the fibre, which seems to contribute.*

*Of Heat and Causticity.* Animal fibres are very sensible to the impressions of heat; a piece of skin or leather, brought near the fire, contracts and moves with some force.† Caustics act on the animal fibres and produce the same

* The author does not include either the elasticity or tone of the animal fibre.

† The author does not take into consideration the same phenomena in vegetables, and between which, as well as their cause, he conceives a very close analogy to exist.

same effects; acids and caustic alkalis crisp and contract the skin; caustics, he says, act by the heat they contain.‡

*Of Moisture and Dryness.* Animal matter, says the author, such as the hair, whale-bone, cat-gut, &c. are very sensible to humidity and dryness; this also exists in vegetables. Glutinous, albuminous, and gummy substances are also affected. The inhabitants of damp climates have the fibre soft, and but little tense; the contrary is the case in dry climates. Moisture and dryness should therefore be considered to act on the excitability of animals.

*Of the particular structure of the fibre as the cause of excitability.* It has been supposed that the nerves were made of vesicles; that these circulated a fluid; that when this fluid was abundant, it swelled these vesicles, and shortened, &c. such as is observed in physics; but anatomy has proved nothing of this kind in the structure of nerves. Reil, who paid much attention to this subject, believes the nerves to be made up of a membrane which forms a number of anfractuosities, in which is deposited the medullary substance.

*Of Galvanism and Electricity.* This is a subject, observes the author, likely to throw much light on the properties of muscles. Galvani observed with astonishment, that the muscles of frogs were thrown into strong contractions when they were touched by metallic substances which also were in contact with their nerves. This experiment has been varied in a thousand shapes. Volta constructed a pile made up of disks of different metals with layers of cloth interposed. He found, 1. That these metals were electrised by simple contact. 2. That this electricity was positive in the one, and negative in the other. 3. That this

‡ We cannot agree with any part of M. De La Melherie's reasoning on the action of heat or caustic on animal matter; the contortions and motions produced on the application of heat, &c. to the skin and leather are owing either to the sudden evaporation of moisture which they contain, or else to a motion arising from decomposition. Heat, after a certain degree, acts on animal matter as it does on common matter, by separating its component particles; and caustics we believe to act by a chemical decomposition similar to what takes place in a variety of other instances, where the principle of irritability is out of the question. The author, in considering heat and caustics as one cause of excitability, has surely mistaken his ground, unless he attaches to the term *excitability*, a meaning different from its general acceptation. That heat acts on excitability, we know, can and does take place; but that it causes it, is a language neither correct nor admissible.

this power was increased by certain substances, as, for instance, by water impregnated with salt. 4. That water was decomposed, and oxygen and hydrogen were disengaged. 5. That the electric spark might be obtained by its means. 6. That metals were oxydated. 7. That this pile produced the same effects on animals as were observed by Galvani.

Humboldt afterwards shewed, that there may be obtained from the frog, Galvanic contractions without the intervention of any metal; and from which he concluded, that nerves had a galvanism different from muscles; that in the body of animals there was a continual passing of galvanic fluid from nerves to muscles, and vice versa. Aldini made this experiment on a larger scale; he separated the head from the trunk of an animal, laid bare the cervical nerves of one side and the muscles of the trunk of the other, and established the communication by means of a frog which he held in his hand. Violent contractions were the consequence.

La Grave made an experiment which, while it confirmed the opinion of Humboldt, throws further light on the question. He formed a pile of disks, alternately, of the substance of the brain and muscle, as in those made of zinc and copper; commotions were in like manner produced. This experiment proves that the substance of the brain is in a state of electricity different from the muscle; but as nerves are dispersed through every part of a muscle, a kind of natural galvanic pile is formed. Vasali had already observed, that the different fluids of the human body were in different states of electricity, and which varied in health and disease. Geoffroy, speaking of the electric organs of the torpedo, says, that its organs are formed of a great number of aponeurotic tubes ranged parallel to each other round the bronchiæ, and fixed at their base by common ligaments of an hexagonal or pentagonal form. These prisms are filled by a soft transparent substance, which receives a great quantity of nerves; this substance, as well as the nerves, is a conductor of electricity, while the aponeurotic tubes are non-conductors. In the gymnotus this apparatus is towards the tail. In the silurus the electric organ forms under the skin a sac which envellopes the entire fish; these apparatus are pierced by a number of nerves, which come from different parts of the body. It appears warrantable to conclude from this, that in all animals the brain furnishes the nerves, and these again the muscles, with a soft pulp, analogous to what is observed observed in fish. This substance is susceptible of Galvanic electricity, as is proved by the experiments of Lagrave Humboldt thinks, that the brain separates the Galvanic fluid. The identity of the electric and Galvanic fluids seem to be sufficiently proved; we must therefore conclude that the brain furnishes the nerves with a nervous pulp containing a great quantity of Galvanic or electric fluid, and that this viscus, so voluminous, filters a particular liquor, which is the nervous, and which also contains a great quantity of Galvanic fluid. When we exercise much, this cerebral pulp loses its Galvanism. A frog, frequently excited, loses its Galvanism, but renews it on resting some time. The reason is, that the nerves and muscles have time to acquire a new quantity of electricity. The means by which these losses are restored, are: 1. Rest; a too long state of rest, observes the author, fatigues, because the electric matter is accumulated in too great quantity. 2. By sleep; this, so much more powerful as the state of rest, is more complete. 3. By moderate heat, which increases the activity of the Galvanic fluid; we know that by heating the cylinder of an electric machine, the effects become more active; in the same way it is that electricity gives energy to the nerves and muscles. 4. Light produces the same effects as heat. 5. By friction, which also promotes Galvanic circulation. The author asks, how is it that Galvanism and electricity produce muscular contraction and excitability? Some physiologists, he remarks, have looked for an explanation of contraction and excitability, by a different combination of animal principles, i. e. hydrogen, oxygen, carbon, azote, phosphorus, &c. Gallini started this opinion, in which he was followed by Gauthier, Vait, Girtanner, &c. Humboldt, who also adopted it, instituted many experiments, with a view to discover the combination which took place during the contraction of fibres. He thinks he can demonstrate that the irritability of animal matter does not depend on the quantity of oxygen which the body contains, but that the azote and hydrogen play at least as important a part, and that the degrees of vitality depend only on the reciprocal balance of chemical affinities. Of all the elements of which animal and vegetable matter is made up, there are certain principles which seem necessary to excite irritability. 1. Oxygen, which enters into different combinations with the acidifiable bases. 2. The acidifiable bases of which the fibre is composed are carbon, azote, hydrogen, phosphorus. The Galvanic fluid favours their combinations, as the electric does that of oxygen and azote azote to form nitre. Let us suppose a fibre composed of a certain number of particles, such as the acidifiable bases, carbon, azote. The arterial blood carries oxygen; the Galvanic fluid, disengaged in passing from the nerve to the muscle, favours the combination of oxygen with the acidifiable bases, a combination takes place and the fibre is shortened, and for this reason it is that no motion takes place where the nerve or artery is tied.*

[ To be continued. ]

* M. Humholdt should have explained the cause which produces the sudden separation of the oxygen, and which we suppose he intends should account for the phenomenon of relaxation.

